# Development and validation of a new predictive model for breast cancer survival in New Zealand and comparison to the Nottingham prognostic index

**DOI:** 10.1186/s12885-018-4791-x

**Published:** 2018-09-17

**Authors:** J. Mark Elwood, Essa Tawfiq, Sandar TinTin, Roger J. Marshall, Tung M. Phung, Ian Campbell, Vernon Harvey, Ross Lawrenson

**Affiliations:** 1 Epidemiology and Biostatistics, School of Population Health, University of Auckland, 261 Morrin Road, Private Bag 92019, Auckland Mail Centre, Auckland, 1142 New Zealand; 2 Waikato Clinical Campus, Department of Surgery, University of Auckland, Hamilton, New Zealand; 3 The University of Waikato, Hamilton, 3240 New Zealand; 4 Waikato District Health Board, Hamilton, New Zealand; 5 Regional Cancer and Blood Centre, Auckland City Hospital, Auckland, New Zealand

**Keywords:** Breast cancer, Mortality, Survival, New Zealand, Predictive model, Prognosis, Nottingham prognostic index

## Abstract

**Background:**

The only available predictive models for the outcome of breast cancer patients in New Zealand (NZ) are based on data in other countries. We aimed to develop and validate a predictive model using NZ data for this population, and compare its performance to a widely used overseas model, the Nottingham Prognostic Index (NPI).

**Methods:**

We developed a model to predict 10-year breast cancer-specific survival, using data collected prospectively in the largest population-based regional breast cancer registry in NZ (Auckland, 9182 patients), and assessed its performance in this data set (internal validation) and in an independent NZ population-based series of 2625 patients in Waikato (external validation). The data included all women with primary invasive breast cancer diagnosed from 1 June 2000 to 30 June 2014, with follow up to death or Dec 31, 2014. We used multivariate Cox proportional hazards regression to assess predictors and to calculate predicted 10-year breast cancer mortality, and therefore survival, probability for each patient. We assessed observed survival by the Kaplan Meier method. We assessed discrimination by the C statistic, and calibration by comparing predicted and observed survival rates for patients in 10 groups ordered by predicted 10-year survival. We compared this NZ model with the Nottingham Prognostic Index (NPI) in this validation data set.

**Results:**

Discrimination was good: C statistics were 0.84 for internal validity and 0.83 for an independent external validity. For calibration, for both internal and external validity the predicted 10-year survival probabilities in all groups of patients, ordered by predicted survival, were within the 95% confidence intervals (CI) of the observed Kaplan-Meier survival probabilities. The NZ model showed good discrimination even within the prognostic groups defined by the NPI.

**Conclusions:**

These results for the New Zealand model show good internal and external validity, transportability, and potential clinical value of the model, and its clear superiority over the NPI. Further research is needed to assess other potential predictors, to assess the model’s performance in specific subgroups of patients, and to compare it to other models, which have been developed in other countries and have not yet been tested in NZ.

**Electronic supplementary material:**

The online version of this article (10.1186/s12885-018-4791-x) contains supplementary material, which is available to authorized users.

## Background

Currently clinicians in New Zealand (NZ) estimate a woman’s likely mortality or survival after a diagnosis of breast cancer (her prognosis) based on experience and clinical judgement. They may use some of the statistical models developed in other countries, such as the Nottingham Prognostic Index (NPI) [1]. A recent review of published data up to July 2012 found 996 articles, from which six prognostic models were identified based on clinical and pathological features [2]. These models were the NPI, (the earliest, published in 1982) [1], Adjuvant! [3], BC Nomogram [4], Options [5], Predict (and Predict+) [6–8], and CancerMath [9]. Validation studies are limited. All these models were developed in European or United States populations, and the few validation studies in other populations show less accurate prediction [10–12]. The only published validation of these models in NZ is a small study involving one of the current authors [13], and no work in Maori or Pacific populations has been done. The models are less accurate in younger and in older patients, e.g., under 40 years and over 75 years [14, 15]. These models use accepted clinical and pathological indicators, such as tumour size, nodal involvement, and receptor status. In addition to these factors, studies in NZ show that there are significant differences in breast cancer outcomes by social deprivation, rural residence, comorbidity, type of health care [16–18]. There are important differences by ethnicity, with Maori and Pacific women being at greater risks of death and recurrence than European NZ women, and these ethnic differences are complex and related to both clinic-pathological and demographic factors [16, 18–20]. Thus we aimed to develop and validate a model, based on NZ patient experience, to predict breast cancer outcomes. If such a model were shown to have acceptable accuracy, it would help clinicians as well as patients and their families, and would facilitate patient-doctor communication and clinical decision making. In this paper, we present a model, the New Zealand Model (NZM), developed to reliably categorise NZ patients into groups by their probability of breast cancer-specific survival within 10 years of an initial breast cancer diagnosis, and compare it to the NPI.

## Methods

### Patients and data

We used the data collected prospectively through the two largest and longest-established population-based regional breast cancer registries in NZ, in the Auckland and Waikato regions. These two regional registries are linked to include over 40% of all patients with breast cancer in NZ, and are representative of NZ women in terms of socioeconomic, demographic and ethnic background [16, 21]. The registries are linked to national mortality data and to the legally-mandated national cancer registry [22] and to other hospital discharge data to assess co-morbidity [22]; comparisons show that the registries are very complete (over 95%) [22]. Mortality and recurrences were documented from regular hospital follow-up, or for patients discharged from regular hospital follow up, from information provided by primary care and private practice physicians, updated annually or more frequently.

The data used are for all women diagnosed with a first primary invasive breast cancer between 1 January 2000 and 30 June 2014, followed up to death or to 31 Dec 2014. Data were missing or incomplete for up to 5% of several items, and 10% for numbers of involved nodes, so we used complete case analysis rather than imputation techniques. There were 10,586 such women in the Auckland registry, of whom 9182 had complete data; their data were used to develop the prediction model and assess its internal validity. Data from the Waikato registry, on 3071 total women and 2625 with complete data, were used to assess the model’s external validity.

### Predictors of breast cancer mortality

Predictors of breast cancer mortality (and therefore of disease specific survival) were selected based on their accepted clinical importance, and then their empirical performance as predictors of 10-year breast cancer mortality [18, 23, 24]. The predictors considered were age at diagnosis, ethnicity, tumour size, number of positive lymph nodes, tumour grade, presence of metastasis at diagnosis (Stage 4), estrogen (ER) and progesterone (PR) receptor status, human epidermal growth factor receptor2 (HER2) status, histological type of tumour, and lymphovascular invasion (LVI). Menopausal status was not retained as it is strongly linked to age. We assessed factors as both continuous and categorical variables, but used categories in the final model. Patient ethnicity was identified from the breast cancer registries or where not available, from the national cancer registry or mortality data following NZ Ministry of Health ethnicity data protocols [25]. Ethnicity was categorized into NZ European, Māori, Pacific, and Other. Cancer stage at diagnosis was defined according to the Tumour, Node, and Metastasis (TNM) system [26]. Invasive tumour grade was defined according to the Elston and Ellis modified Scarff-Bloom-Richardson breast cancer grading system [27]. Estrogen (ER) and progesterone (PR) receptor status was based on the results of immunohistochemistry tests and classified as positive with 1% or more receptor positive cells [28], and grouped as both ER and PR positive, both negative, and either positive/negative or negative/positive status. HER-2 status was based on a Fluorescent In-Situ Hybridization (FISH) test or when this was not available, on immunohistochemistry [29], and categorised as positive, negative/equivocal, or not tested. HER2 assessment was introduced only in 2006.

### Statistical methods

We performed Cox multivariate proportional hazards regression analysis. The outcome variable was defined as time from diagnosis until death due to breast cancer, with censoring at date of death from other causes, or 31 Dec 2014. Analyses were performed with SAS version IC.11 [30] and R version 3.2.5 [31].

The New Zealand model (NZM) was built using the Auckland data base. Various models were developed using continuous or categorical variables, with an improvement in goodness-of-fit assessed by a reduction in the Akaike Information Criterion (AIC) [32]. The models fit a mortality function, S(t) as the probability of mortality for time *t*, dependent on S_0_(t), the baseline mortality probability for time t, and Xβ, the linear combination of predictors of breast cancer mortality [32]. We obtained the estimated 10-year mortality probability at baseline, by setting the predictors to their reference levels and fitting the Cox multivariate regression. Then we used the mortality function [32], and computed 10-year mortality for each patient in the Auckland database. We tested the proportional hazards assumption using graphical log-log survival plots and the method of weighted residuals [33], and we tested for goodness-of-fit using the procedure of May and Hosmer [34].

We fitted a model with continuous variables of age, tumour size, and number of positive lymph nodes, and categorical variables of ethnicity, tumour stage, tumour grade, ER and PR receptors, HER2 status, histological type of tumour, and LVI. Then we fitted the model with categorical variables of all the predictors, and found that the fit improved, omitting LVI which was no longer significant (*p* > 0.05). Graphical representation of the model was done with a regression nomogram, enhanced with distribution of covariates shown by scaled-to-frequency boxes [35] and produced with R function regplot [36].

We assessed internal validity using the Auckland database. Internal validity was assessed with bootstrapping (200 replications). Bootstrapping samples were created by drawing random samples with replacement from the Auckland database. To assess discrimination, we used the C statistic [32]. The prediction model was fitted on each bootstrap sample and tested on the original sample. To assess calibration, we divided patients in the Auckland database into ten groups, ordered by their predicted 10-year breast cancer survival. We then compared for each group the mean of the predicted 10-year breast cancer survival with the observed 10-year breast cancer survival [32, 37] calculated by the Kaplan-Meier method [38].

To assess external validity and transportability of the model, we applied the Auckland-developed model to an independent data set, the Waikato registry. External validity of the model was assessed by bootstrapping (200 replications), using the Waikato database. For the Waikato data, we assessed the C statistic, and compared predicted and observed 10-year breast cancer survival in groups ordered by predicted survival. Since there were few patients with predicted survival under 30%, we combined patients with 10-year breast cancer survivals of 0–30% in one group, leaving 8 groups.

There are no simple calculations of statistical power in predictive models, but assessing external validity, a minimum of 100 events has been recommended for mortality analysis [39, 40]. Another study suggests a minimum of 10 events per predictor for proportional hazards regression [41]. We estimated that in the smaller external validation data registry there were 282 breast cancer specific deaths, giving 31 events per predictor variable, which was adequate.

### Comparison with NPI

The NPI was calculated for patients in the Waikato data, based on tumour size, pathological grade, and number of positive nodes [42], which replaces nodal stage used originally [1], as in other validation studies of the NPI [43, 44]. In keeping with the development of the NPI, only patients with stage 1–3 breast cancer, and tumour size> 0 cm were included [1]. Thus 46 patients with Stage IV tumours and 2 with missing tumour grade were excluded, so the comparison was done in 2579 patients. Following NPI methods, we classified patients into three NPI prognostic groups, defined as good (NPI < 3.4), moderate (3.4 to 5.4), and poor (NPI > 5.4) [45]. Within these subgroups, subdivided by deciles of breast cancer-specific survival predicted by the NZ model, we compared the predicted and observed breast cancer-specific mortality. The mortality probability predicted by the NZ model in a subgroup was the mean of all the predicted probabilities generated for all patients in that subgroup. The model was considered accurate if the predicted outcome was within the 95% confidence interval (95%CI) of the observed outcome. An a priori alpha level of 0.05 was used. The difference of means of breast cancer-specific mortality predicted by the NZM in the three NPI groups was tested using one-way ANOVA. Because of the unequal sample size between the groups, a Tukey post hoc test was used for pairwise comparisons of means [46].

## Results

For the 9182 eligible women in the Auckland database, there were 864 breast cancer specific deaths over the 14-year time period; median follow up time was 67.6 months, and mean age of patients 56.9 years (Table 1). Patients were predominantly Stage 1 (43%) and 2 (39%), ER and PR positive (79 and 68%), HER-2 negative (69%), without lymphovascular invasion (73%), and with ductal tumours (81%). Of the patients, 71% were of NZ European ethnic group, with 8% Maori, 7% Pacific, and 14% other (such as Asian countries).

**Table 1:** Features of patients included in the derivation data set (Auckland) and the independent validation data set (Waikato)

	Derivation (Auckland)	Validation (Waikato)
Total number of women with invasive cancer	10,586	3071
Number of complete cases	9182	2625
Number of deaths due to breast cancer	864	282
Median follow-up time (in months)	67.6	68.4
Age (mean in years)	56.9	59.3
Tumour grade		
Well differentiated	2229 (24%)	624 (24%)
Moderately differentiated	4108 (45%)	1406 (53%)
Poorly differentiated	2845 (31%)	595 (23%)
Tumour size (mean in mm)	23.9	22.4
Mean number of positive lymph nodes removed	2.4	1.9
Stage of tumour		
Stage 1	3990 (43%)	1071 (41%)
Stage 2	3602 (39%)	1117 (42%)
Stage 3	1432 (16%)	391 (15%)
Stage 4 (presence of metastasis)	158 (2%)	46 (2%)
ER status		
Negative	1914 (21%)	410 (16%)
Positive	7268 (79%)	2215 (84%)
PR status		
Negative	2919 (32%)	903 (34%)
Positive	6270 (68%)	1722 (66%)
HER2 status		
Positive	1156 (13%)	378 (14%)
Negative/equivocal	6331 (69%)	1750 (67%)
Test not done	1695 (18%)	497 (19%)
Histological type of tumour		
Ductal	7469 (81%)	2130 (81%)
Lobular	1072 (12%)	294 (11%)
Mixed	641 (7%)	201 (8%)
Lymphovascular invasion status		
Negative	6730 (73%)	1939 (74%)
Positive	2452 (27%)	686 (26%)
Ethnicity		
Maori	720 (8%)	388 (15%)
Pacific	682 (7%)	40 (2%)
European NZ	6527 (71%)	2131 (81%)
Other	1253 (14%)	66 (2%)

Data collection on HER2 status started in 2006

The predictors in the final model are shown in Table 2. The risk of breast cancer mortality within 10 years of diagnosis increased significantly with age being over 70 years; higher tumour grade, larger tumour size, greater number of positive lymph nodes, presence of metastases at diagnosis, and with ER or PR negative tumours. Mortality risk was reduced with HER2 status positive, and histological types other than ductal or lobular. NZ European, Maori, and Pacific women had similar risks of mortality, but it was lower in patients in the ‘Other’ ethnic group. When tested for proportional hazards, most of the covariates met the criterion, but overall, the global criterion was not met. Nevertheless when the model was tested for goodness-of-fit [34] it was found to be an adequate fit (*p* = 0.26).

**Table 2:** Predictors of 10 year breast cancer mortality, Auckland data, 1 Jan 2000–31 Dec 2014

Risk factor	Coefficient	SE	Hazard ratio (HR)	95% confidence limits	*p*-value
Age under 40	0.085	0.122	1.09	0.86–1.38	0.486
Age (40–49)	−0.003	0.097	1.00	0.83–1.21	0.976
Age (50–59)			Ref		
Age (60–69)	−0.048	0.106	0.95	0.78–1.17	0.652
Age 70 & over	0.388	0.104	1.47	1.20–1.81	< 0.001
Tumour grade 1			Ref		
Tumour grade 2	1.103	0.201	3.01	2.03–4.46	< 0.001
Tumour grade 3	1.504	0.206	4.50	3.01–6.73	< 0.001
Tumour size (0.1–19.9 mm)			Ref		
Tumour size (20–49.9 mm)	0.734	0.088	2.08	1.75–2.48	< 0.001
Tumour size (50 mm & more)	1.047	0.118	2.85	2.26–3.59	< 0.001
Positive lymph nodes (zero node)			Ref		
Positive lymph nodes (1–3 nodes)	0.783	0.094	2.19	1.82–2.63	< 0.001
Positive lymph nodes (4–9 nodes)	1.277	0.106	3.59	2.92–4.41	< 0.001
Positive lymph nodes (10 nodes & more)	1.722	0.111	5.60	4.50–6.96	< 0.001
Presence of metastases at diagnosis	1.603	0.120	4.97	3.93–6.28	< 0.001
No metastases at diagnosis			Ref		
Hormone receptor (1 negative & 1 positive)	0.636	0.096	1.89	1.56–2.28	< 0.001
Hormone receptor (double negative)	1.023	0.091	2.78	2.33–3.32	< 0.001
Hormone receptor (double positive)			Ref		
HER2 status (positive)	−0.206	0.094	0.81	0.68–0.98	< 0.05
HER2 status (test not done)	0.164	0.088	1.18	0.99–1.40	0.061
HER2 status (negative/equivocal)			Ref		
Lobular histological type of cancer	−0.046	0.121	0.95	0.75–1.21	0.702
Other histological type of cancer	−0.576	0.206	0.56	0.38–0.84	< 0.01
Ductal histological type of cancer			Ref		
Maori	0.121	0.121	1.13	0.89–1.43	0.316
Pacific	0.007	0.118	1.01	0.80–1.27	0.949
Other	−0.518	0.124	0.60	0.47–0.76	< 0.001
NZ European			Ref		

**Fig. 1:**
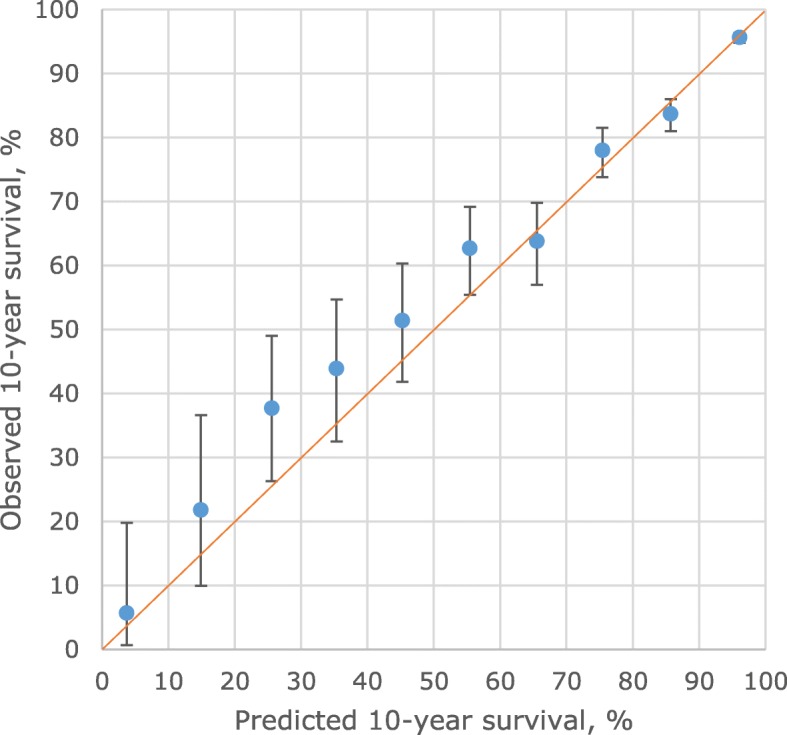
Internal validity: 10-year breast cancer specific survival as predicted by the NZ model (horizontal axis) for 10 groups of patients in the derivation data set (Auckland), grouped by predicted survival,compared to observed (Kaplan-Meier) survival and its 95% confidence limits (vertical axis). Line of identity between predicted and observed survival shown

Fitting the model to the Auckland data, assessing internal validity, gave a C statistic of 0.84. Calibration is shown as survival rather than mortality as that is usually used clinically, comparing predicted and observed 10-year disease specific survival (1-mortality). Figure 1 (and Additional file 1: Table S1) show that for patients divided into ten groups, based on predicted 10-year breast cancer survival of 0–9%, 10–19%, etc., the predicted survivals were within the 95% confidence interval (CI) of observed survival for all groups.

**Fig. 2:**
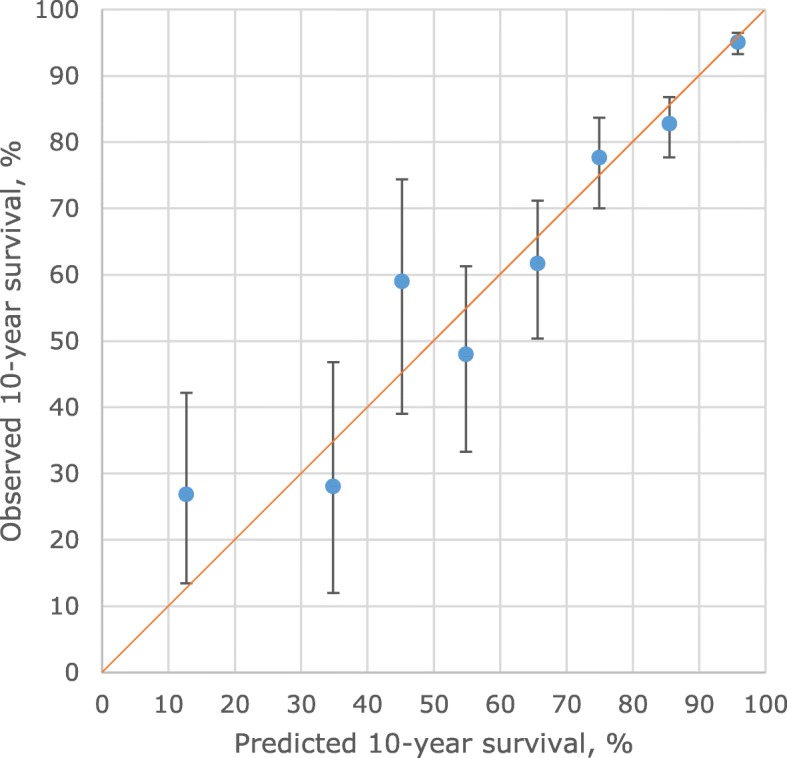
External validity: 10-year breast cancer specific survival as predicted by the NZ model (horizontal axis) for 8 groups of patients in the validation data set (Waikato), grouped by predicted survival, using Auckland derived model, compared to observed (Kaplan-Meier) survival and its 95% confidence limits (vertical axis). Line of identity between predicted and observed survival shown

To assess external validity, the model developed in the Auckland data was applied to the independent data set, 2625 patients in the Waikato registry, with 282 deaths. These patients were of similar mean age to those in Auckland, but with relatively more Maori, fewer Pacific, and fewer of ‘other’ ethnic background (other than Maori, Pacific, and European NZ). The distributions of clinico-pathological factors was generally similar (Table 1). The Auckland-derived model showed good discrimination in this independent data set, with the C statistic being 0.83. For calibration (Fig. 2), from 10 groups ordered by predicted survival, the three groups with the lowest survival had few patients and were combined into a 0–30% group. In all the eight groups, the predicted 10-year breast cancer survivals were within the 95% confidence interval of the observed 10-year breast cancer survival.

**Fig. 3:**
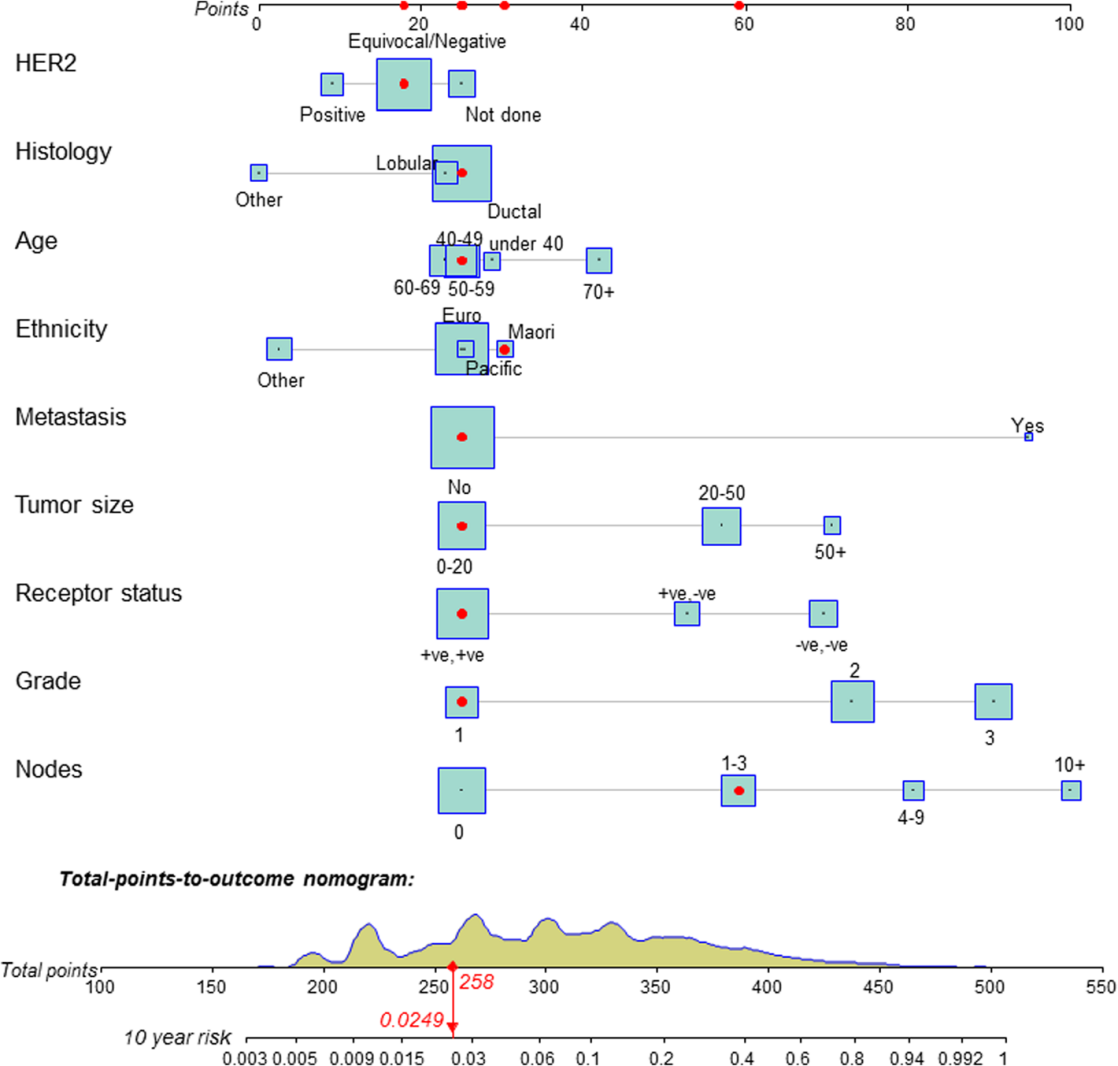
A nomogram of the fitted model, showing the relative contribution of variables to the model and also, by relative sizes of the boxes, the distribution of each. The distribution of the total score is also shown and the dots show a particular person with 10-year mortality risk of 2.5% (95% CI 1.3–3.6%)

Representation of the final model by a nomogram is shown in Fig. 3. It is ranked showing the greatest contribution to the regression from top downwards. The figure also shows the total score, and its distribution, and 10-year (120 month) risk for a person with the indicated set of predictors (2.5% estimated mortality risk).

The NPI was also applied to the independent data set, dividing patients into three prognostic groups (Table 3). While the mean predicted 10-year breast cancer survival showed significant differences and a trend, from 96.1% in ‘good’, 84.0% in ‘moderate’, to 57.8% in the ‘poor’ NPI groups, the range of predictions for individual patients was very large within each group. Within each of these NPI subgroups, the NZ model shows good discrimination and predictive ability (Table 4). In all assessable subgroups, the predicted breast cancer deaths were within the 95% CI of the observed deaths. Within the ‘good’ prognosis NPI group, patients fall into the highest 8 of the 10 deciles by predicted survival using the NZ model, with predicted survival rates ranging from 80 to 99%. Within the NPI ‘poor’ prognosis group, all but 3 patients are in the lowest 5 deciles, with predicted survival rates ranging from 42 to 88%.

**Table 3:** 10-year survival predicted by the NZ model in three NPI prognostic groups

	Good prognostic group	Moderate prognostic group	Poor prognostic group	*P*-value
Number of patients (%)	1021 (39.6)	1123 (43.5)	435 (16.9)	
Mean ± SD	96.12 ± 3.21	84.04 ± 10.35	57.79 ± 19.85	< 0.001
Range (Min-Max)	18.46 (81.15–99.61)	63.84 (34.65–98.49)	90.56 (3.45–94.01)	

each pairwise comparison of means was significant (*P* < 0.001)

**Table 4:** Observed and predicted breast cancer survival by NPI prognostic groups and decile of risk predicted by the NZ model

Decile of NZ index		NPI good prognostic group					NPI moderate prognostic group						NPI poor prognostic group					
	Total cases	Number of predicted deaths	Number of observed deaths (KM)	Predicted survival rate NZ model	Observed survival rate, KM	95% CI observed survival	Total cases	Number of predicted deaths	Number of observed deaths (KM)	Predicted survival rate NZ model	Observed survival rate, KM	95% CI observed survival	Total cases	Number of predicted deaths	Number of observed deaths (KM)	Predicted survival rate NZ model	Observed survival rate, KM	95% CI observed survival
1	259	2.6	0.0	99.0	100.0		0	0	0	100.0			0	0	0			
2	239	4.8	6.4	98.0	97.3	91.2–99.2	18	0.4	0	97.8	100.0		0	0	0			
3	211	8.3	7.2	96.1	96.6	90.9–98.7	60	2.2	1.5	96.3	97.5	83.5–99.7	0	0	0			
4	156	8.3	6.5	94.7	95.8	87.4–98.7	88	4.9	2	94.4	97.7	85.2–99.7	1	0.1	1	90.0	0.0	
5	76	6.3	5.5	91.7	92.8	73.9–98.2	186	14.8	23	92.0	87.6	77.6–93.3	2	0.1	0	95.0	100.0	
6	64	6.6	6.0	89.7	90.6	76.3–96.4	178	19.3	28.1	89.2	84.2	74.8–90.3	11	1.3	0	88.2	100.0	
7	13	2.2	3.7	83.1	71.5	26.2–92.3	218	33.6	21.3	84.6	90.2	82.5–94.7	26	4	2.9	84.6	88.8	61.5–96.9
8	3	0.6	0.0	80.0	100.0		188	40.2	39.3	78.6	79.1	71.0–85.2	69	15.8	12.4	77.1	82.0	65.7–91.2
9	0						143	43.1	29.6	69.9	79.3	68.1–86.9	114	39.1	31.4	65.7	72.5	61.3–81.0
10	0						44	20.7	10.8	53.0	75.5	51.6–88.6	212	123.2	119.6	41.9	43.6	34.5–52.4

## Discussion

In this study we used demographic, clinical and pathologic factors to build a statistical model, the NZ model, to estimate the probability of breast-cancer specific survival, or equivalently death, within 10 years of diagnosis in women diagnosed with primary invasive breast cancer in NZ. Our results confirmed that many factors affect a woman’s prognosis; age at diagnosis, number of positive lymph nodes, tumour size, tumour grade, presence of metastases at diagnosis, histological type of tumour, ER and PR receptors status, and HER2 status, all had significant associations with breast cancer specific survival. These factors have been used to predict breast cancer outcomes by several studies [4, 6, 7, 9, 14, 17, 47].

In terms of validity and reliability, we found that our predictive model performed well even in external validation on an independent data set. The C statistic was 0.83 for independent validation, compared to 0.84 for internal validation in the data from which it was derived, showing good discrimination [37]. Calibration assessment indicated good agreement between predicted and observed 10-year breast cancer specific survival. In both internal and external validation,predicted survivals were within the 95% CI of observed survival probabilities in all groups of patients. These are accepted approaches for assessing discrimination and calibration of prediction models of breast cancer outcomes [7, 14, 32, 37, 47]. Power was satisfactory: we had 282 breast cancer specific deaths, and 31 events per predictor variable in the smaller validation cohort, compared to recommendations of 100 events [39, 40], and 10 events per predictor variable being adequate [41]. In future work, we will explore the model’s performance in further groups of patients, and ultimately its validity at the individual patient level.

The optimum presentation of the results of any model is a complex issue. In Fig. 3 the model is represented by a regression nomogram; this way of representing risk models is becoming common and has been recommended [48]. The nomogram is enhanced with covariate and total distributions. When active on a computer it can present interactive calculations of individuals’ risks. We see the applications of our prognostic model in assisting informed decision-making by women with breast cancer, their doctors and their carers. In further research we will assess the most effective ways to use the prognostic estimates. Many current models use overly complex language in the presentation of results, such that one study found that fewer than half of patients understood their prognosis after an oncologist’s consultation [49]. Health literacy issues need to be considered as they contribute to health inequities [50, 51].

There are few comparisons of more than one risk prediction model applied to the same independent patient population. A recent study showed similar performance of three models in patients recorded in an international tissue bank, but these patients are not representative of all incident patients [47]. A 2009 review [52] concluded that the Nottingham index has been the most tested, and only two other models had any published validation in an independent population. Most validation studies have used 1000 patients or fewer [2]. Several other models are based on genetic profiles or novel biomolecular factors, e.g. Oncotype DX [11, 53], but not also including clinical and pathological factors.

In New Zealand, the patient’s ethnicity, with the three largest groups being NZ European, Maori, and Pacific, is of great general importance, so we kept ethnicity in the prognostic model. Maori and Pacific women in NZ with breast cancer have a worse prognosis and higher mortality [23, 54], however, the current results show that Maori or Pacific ethnicity have no independent predictive value on 10-year survival, once detailed clinical and pathological factors are taken into account. This suggests the ethnicity factors are mediated through these clinical and pathological variables, and agrees with other detailed analyses [16]. Thus, while at the population level different approaches may be needed to overcome the disparities in outcomes of the different ethnic groups, at a clinical level the assessment of prognosis in each women may not be affected by her ethnic group. Thus, for example, in women whose breast cancers have been screen-detected, outcomes are equivalent in Maori and non-Maori women in NZ [55]. However, there may be ethnic differences in total mortality and in morbidity or recurrence, so in further work we will assess whether ethnic-specific prognostic models have any advantages.

Among the strengths of our modelling process is that we have used large datasets for both development (over 9000 patients) and validation (over 2600 patients), and both are population-based, including virtually all diagnosed patients [22]. These women have undergone routine clinical treatment and their survival experience will reflect this and depend on their diagnosis date, from 2000 to 2014. As one comparison, the UK developed ‘Predict’ model was based on 5738 patients with complete data diagnosed from 1999 to 2003 [8], but has been shown to be applicable to several other populations. All predictive models based on actual patient experience will be limited by the assessment and treatments available at the time they were diagnosed.

We developed our model based on the patients with data available on all relevant factors, as in its application to new patients all information is likely to be available as it can be actively sought. Incomplete data were more common in patients with more advanced disease; probably because documentation of features of the primary disease such as tumour size or number of involved nodes is less relevant clinically in these patients. It implies that the prognostic model may be less accurate in patients with metastatic disease. For patients with some missing data, associations between known factors and 10-year survival were generally similar to those of patients with complete data.

We did not include treatment variables in our models. The models are derived from data on the experience of unselected population-based series of breast cancer patients. Their treatment will have been guided by international best practice, summarised in NZ clinical guidelines [56] and standards of service [57]. Many patients may not receive the standard recommended treatment for various reasons including patient choice, comorbidity, and barriers to access of health care. In future work, we will explore effects of treatment factors, both in regard to not receiving recommended treatments, and also receiving new treatments; to do this, we will include in the model estimates of treatment effects, where possible based on the results of randomised trials.

The NPI was the first breast cancer prognostic model published [1], has had the most extensive validation [2], and is still widely used. It does not predict survival for each patient, but divides patients into prognostic groups, usually three groups. It is based on three factors incorporated in the NZ model and so the two indices are related. We have shown here that the NPI is only very approximate, there being wide variations in survival within each NPI prognostic group. We have demonstrated that within each NPI group the NZ model subdivides patients into smaller groups efficiently, with good correlation between predicted and observed ten-year breast cancer specific survival rates. The NZ model could replace the NPI in those situations were the NPI is being used.

We will do further work to compare our model’s performance with that of other available models, developed in other countries. We will also assess other potential predictive factors and their effects on the performance of our model. These will include treatments, and comorbidity to account for associated health conditions of breast cancer patients. We will also assess other outcomes of breast cancer, such as overall mortality, local recurrence rate, and recurrence rate.

## Conclusions

We have developed a NZ specific predictive model, which has good validity to predict breast cancer mortality in women with primary invasive breast cancer in NZ. The model is clearly superior to the widely used NPI, and categorises patients by predicted survival even within categories of the NPI. The NZ model shows potential to have important clinical value.

## Additional file

Additional file 1: **Table S1**. 10-year breast cancer predicted and observed survival, Auckland and Waikato databases. (DOCX 14 kb)

## Abbreviations

ER: Estrogen receptor
FISH: Fluorescent in-situ hybridization
HER-2: Human epidermal growth factor receptor 2
HR: Hazard ratio
KM: Kaplan-meier
LVI: Lympho-vascular invasion
NPI: Nottingham prognostic index
NZ: New Zealand
NZM: New Zealand model
PR: Progesterone receptor
TNM: Tumour, Node, Metastasis
