# Supplementary material for: Development and validation of a new predictive model for breast cancer survival in New Zealand and comparison to the Nottingham prognostic index
Source: BMC Cancer. 2018 Sep 17;18:897. doi: 10.1186/s12885-018-4791-x (PMC6142675; doi:10.1186/s12885-018-4791-x)
Supplement: Supplementary file 1 — Table S1. 10-year breast cancer predicted and observed survival, Auckland and Waikato databases. (DOCX 14 kb) [file 12885_2018_4791_MOESM1_ESM.docx]

| Supplementary Table 1. 10-year breast cancer predicted and observed survival, Auckland and Waikato databases | | | | | | | |
| --- | --- | --- | --- | --- | --- | --- | --- |
| Regions | Groups, based on predicted survival | Number of patients | Number of deaths | Predicted | Observed | | |
|  |  |  |  | Survival (mean) | Survival | Lower limit | Upper limit |
| Auckland |  |  |  |  |  |  |  |
|  | 0.0-0.1 | 76 | 50 | 4 | 6 | 1 | 20 |
|  | 0.1-0.2 | 78 | 43 | 15 | 22 | 10 | 37 |
|  | 0.2-0.3 | 108 | 49 | 26 | 38 | 26 | 49 |
|  | 0.3-0.4 | 122 | 49 | 35 | 44 | 33 | 55 |
|  | 0.4-0.5 | 194 | 67 | 45 | 51 | 42 | 60 |
|  | 0.5-0.6 | 313 | 81 | 55 | 63 | 55 | 69 |
|  | 0.6-0.7 | 437 | 98 | 66 | 64 | 57 | 70 |
|  | 0.7-0.8 | 785 | 115 | 75 | 78 | 74 | 82 |
|  | 0.8-0.9 | 1657 | 165 | 86 | 84 | 81 | 86 |
|  | 0.9-1.0 | 5412 | 128 | 96 | 96 | 95 | 96 |
| Waikato |  |  |  |  |  |  |  |
|  | 0.0-0.3 | 47 | 30 | 13 | 27 | 13 | 42 |
|  | 0.3-0.4 | 29 | 18 | 35 | 28 | 12 | 47 |
|  | 0.4-0.5 | 40 | 13 | 45 | 59 | 39 | 74 |
|  | 0.5-0.6 | 88 | 29 | 55 | 48 | 33 | 61 |
|  | 0.6-0.7 | 138 | 37 | 66 | 62 | 50 | 71 |
|  | 0.7-0.8 | 204 | 35 | 75 | 78 | 70 | 84 |
|  | 0.8-0.9 | 519 | 55 | 85 | 83 | 78 | 87 |
|  | 0.9-1.0 | 1560 | 41 | 96 | 95 | 93 | 97 |
